# Modeling epigenetic regulation of PRC1 protein accumulation in the cell cycle

**DOI:** 10.1186/s13062-015-0078-1

**Published:** 2015-10-12

**Authors:** Marzena Dolbniak, Marek Kimmel, Jaroslaw Smieja

**Affiliations:** Systems Engineering Group, Silesian University of Technology, Akademicka 16, 44-100 Gliwice, Poland; Departments of Statistics and Bioengineering, Rice University, MS 138, 6100 Main, Houston, TX 77005 USA

**Keywords:** Cell cycle, Mathematical model, PRC1 protein, Dynamics, Stochastic fluctuations, Asymmetric division

## Abstract

**Background:**

Epigenetic regulation contributes to many important processes in biological cells. Examples include developmental processes, differentiation and maturation of stem cells, evolution of malignancy and other. Cell cycle regulation has been subject of mathematical modeling by a number of authors that resulted in many interesting models and application of analytic techniques ranging from stochastic processes to partial differential equations and to integral, functional and operator equations. In this paper we address the question of how the regulation of protein contents influences the long-term dynamics of the population. To accomplish this, we follow the philosophy of a 1984 model by Kimmel et al., but adjust the details to fit the experimental data on protein PRC1 from a more recent paper.

**Results:**

We built a model of cell cycle dynamics of the PRC1 and fitted it to the data made available by Cohen and his co-authors. We have run the model for a large number of cell generations, recording the PRC1 contents in all cells of the resulting pedigree, at constant time intervals. During cell division the PRC1 is unequally divided between daughter cells. The picture emerging from simulations of Data set 1 is that of a very well-tuned regulatory circuit that provides a stable distribution of PRC1 contents and interdivision times. Data set 2 seems qualitatively different, with more variation in cell cycle duration.

**Conclusions:**

The main question we address is whether the regulatory feedbacks deduced from single cell cycle data provide epigenetic regulation of cell characteristics in long run. PRC1 is a good candidate because of its role in setting timing of division. Findings of the current paper include tight regulation of the cell cycle (particularly the timing of the cell cycle) even that PRC1 is only one of the players in cell dynamics. Understanding that association, even close, does not necessarily imply causation, we consider this an interesting and important result.

**Reviewers:**

This article was reviewed by Ollivier Hyrien, Anna Marciniak-Czochra and Alberto d’Onofrio.

## Background

Epigenetic regulation contributes to many important processes in biological cells. Examples include developmental processes, differentiation and maturation of stem cells, evolution of malignancy and other [1]. One of the processes, which have been studied for at least several decades, is regulation of cell size and cell cycle duration. More specifically, how the dynamics of protein production and the manner in which proteins are split between the two progeny cells leads to preservation of cell population age and size structure (homeostasis). A related question is under what circumstances these dynamics lead to phenomena such as bimodality and, as a consequence, separation of distinct cell subpopulations.

Cell cycle regulation has been subject of mathematical modeling by a number of authors which resulted in many interesting models and application of analytic techniques ranging from stochastic processes to partial differential equations and to integral, functional and operator equations [2, 3]. Some of the models have been applied to data on bacterial and eukaryotic cells, also in the context of cancer modeling [4].

**Fig. 1:**
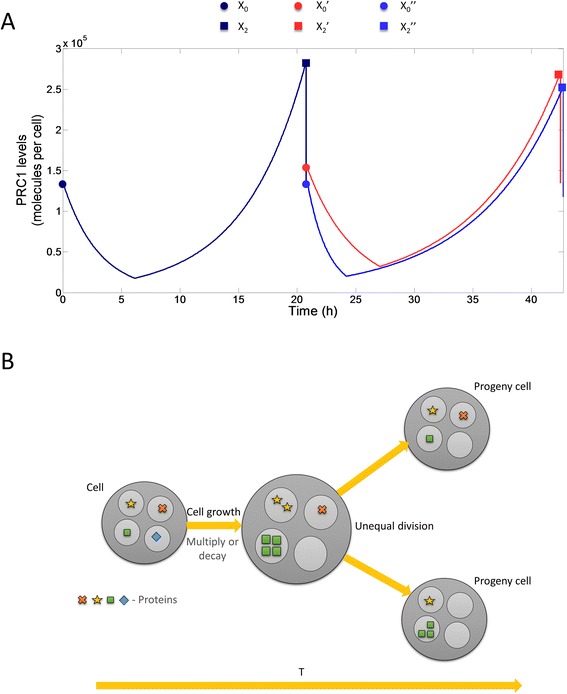
Schematics of the models of asymmetric division. **a** Model of Kimmel et al. (1984). Birth-mass of a cell, represented by random variable (rv) *X*_0_, determines both the mass at division, *X*_2_, and the time, *T*, to division (cell-cycle duration): which considers the dynamics of the distribution of the total mass of cell RNA in a growing cell population. In that model, the birth-mass of a cell, represented by random variable (rv) *X*_0_, determines both the mass at division, *X*_2_, and the time, *T*, to division (cell-cycle duration): *X*_2_ = *ϕ*(*X*_0_), *T* = *ψ*(*X*_0_). At division, the parent-cell mass is randomly split between the two progeny cells, according to the expression *X*_0_ ' = *UX*_2_, *X*_0_ ' ' = (1 − *U*)*X*_2_ in which the random variable *U* is independent of rv *X*_2_, and it is distributed symmetrically over the interval (0, 1), so that *E*(*U*) = 1/2. Uneven partition of mass among progeny cells is the only source of randomness in the basic model. **b** Model of Kimmel (1997). Large particles (biological cells), follow a binary fission process. Each of the large particles is born containing a number of small particles (genes, proteins, viruses, organelles), which multiply or decay during the large particles lifetime. Small particles are then split between the two progeny of the large particle and the process continues in each of them

An example of an early model devised to capture cell cycle regulation and unequal division of mass among progeny cells, is the model by Kimmel et al. [5] (Fig. 1), which considers the dynamics of the distribution of the total mass of cell RNA in a growing cell population. In that model, the birth-mass of a cell, represented by random variable (rv) *X*_*0*_, determines both the mass at division, *X*_*2*_, and the time, *T*, to division (cell-cycle duration).

1 $$ {X}_2=\phi \left({X}_0\right),\kern0.5em T=\psi \left({X}_0\right) $$

At division, the parent-cell mass is randomly split between the two progeny cells, according to the expression

2 $$ {X}_0\hbox{'}=U{X}_2,\kern0.5em {X}_0"=\left(1-U\right){X}_2 $$

in which the rv *U* is independent of rv *X*_2_, and it is distributed symmetrically over the interval (0, 1), so that *E(U) = 1/2*. Uneven partition of mass among progeny cells is the only source of randomness in the basic model. A more general version of the model retains the deterministic mass growth, but includes stochastic time to division [6]. In other models, the growth of mass is deterministic or stochastic and division occurs when a randomly assigned mass threshold is reached [3]. The model leads to stable exponential growth, a process in which the numbers of cells in all possible subsets of (*X*_0_, *X*_2_, *T*) values grow exponentially at a rate defined by a Malthusian parameter *λ*. Please see the Conclusions Section for further remarks.

Cell-to-cell differences are present in any cell population. The sources of variation in population include the extrinsic and intrinsic noise and are well characterized for many cell types (e.g., [7, 8]). Non-genetic intrinsic heterogeneity stems from the random (thermal) nature of interaction of individual molecules, such as mRNA and proteins. Since some of these biomolecules are present in a relatively small number in a cell, their stochastic fluctuations are, unlike in classical test-tube chemistry, not averaged out [1]. As stated above, an equally important source of heterogeneity is the unequal distribution of cellular mRNA and proteins between two daughter cells after cell division.

In this paper we address the question of how the regulation of protein contents influences the long-term dynamics of the population. To accomplish this, we follow the philosophy of Kimmel et al. model [5], but adjust the details to fit the experimental data on protein PRC1 from the paper by Cohen et al. [9]. PRC1 protein is expressed at relatively high levels during S and G2/M phases of the cell cycle before dropping dramatically after mitotic exit and entrance into G1 phase. PRC1 is a substrate of several cyclin-dependent kinases (CDKs) and it has become a novel human protein of cytokinetic importance since its identification [10]. PRC1 takes part in midzone microtubule formation and is essential to the cytokinetic machinery of mammals, via collaboration with Kinesin-4 in setting up a controlled zone of overlapping, antiparallel microtubules at the spindle midzone [11]. Upon anaphase onset and removal of inhibitory CDK1 phosphorylation, PRC1 dimers form, which recruit Kinesin-4, a plus-end directed motor protein that inhibits microtubule dynamics, helps stabilize and regulate spindle microtubule assembly within cytokinesis. The PRC1-Kinesin-4 complex identifies and regulates the spindle midzone microtubules during cell division, which is crucial in order for cytokinesis to progress properly. Our model assumes that PRC1 dynamics contributes to determination of the duration of the cell cycle, with influence of other factors represented as noise.

Further information concerning the role of PRC1 is found in the papers [12–16]: The role of PRC1 in cancer has been considered in references [12, 17] and the role in radiation resistance and stemness in reference [18].

## Methods

### Hypotheses

As already mentioned, the model we use is patterned after the model by Kimmel et al. [5]. Dynamics of PRC1 during a single cell cycle are separated into two phases: degradation and accumulation. The following variables are used to describe the two phases:

*X*_0_ – the number of PRC1 molecules at the beginning of the cell cycle,

*X*_1_ – minimum number of molecules, at the end of the degradation phase and the beginning of the accumulation phase,

*X*_2_ – number of molecules at the end of the cell cycle,

*T*_1_ - duration of the degradation phase,

*T*_2_ – duration of the accumulation phase,

*a* – protein degradation rate, and

*b* – protein production rate.

We assume that in the degradation phase the dynamics of the protein are described as exponential decay, while in the accumulation phase they are described as exponential growth

3 $$ {X}_1={X}_0 \exp \left(-a{T}_1\right),\kern1em {X}_2={X}_1 \exp \left(b{T}_2\right) $$

We assume that following cell division, the protein is split unequally among the two progeny cells according to expressions (2). In the balanced exponential growth, *X*_0_, *X*_2_, and *U* are distributed identically in each cell and so we have *X*_0_ = *UX*_2_ which by independence of *X*_2_ and *U* implies

$$ V\left({X}_0\right)=E\left({X}_2^2\right)E\left({U}^2\right)-E{\left({X}_2\right)}^2E{(U)}^2=V\left({X}_2\right)E\left({U}^2\right)+E{\left({X}_2\right)}^2V(U) $$

$$ \frac{V\left({X}_0\right)}{E{\left({X}_0\right)}^2}=\frac{V\left({X}_2\right)E\left({U}^2\right)}{E{\left({X}_0\right)}^2}+\frac{E{\left({X}_2\right)}^2V(U)}{E{\left({X}_0\right)}^2} $$

Considering that because of symmetry *E*(*U*) = 1/2 and *E*(*X*_0_) = *E*(*X*_2_)/2 we obtain

$$ \frac{V\left({X}_0\right)}{E{\left({X}_0\right)}^2}=4\frac{V\left({X}_2\right)E\left({U}^2\right)}{E{\left({X}_2\right)}^2}+4V(U) $$

and passing to the coefficients of variation ($c{v}_X=\sqrt{V(X)}/E(X)$ for rv *X*), we obtain

$$ c{v}_{X_0}^2=c{v}_U^2\left(c{v}_{X_2}^2+1\right)+c{v}_{X_2}^2 $$

Solving the above for *cv*_*U*_^2^ results in

4 $$ c{v}_U^2=\frac{c{v}_{X_0}^2-c{v}_{X_2}^2}{c{v}_{X_2}^2+1} $$

### Data-based model building

**Fig. 2:**
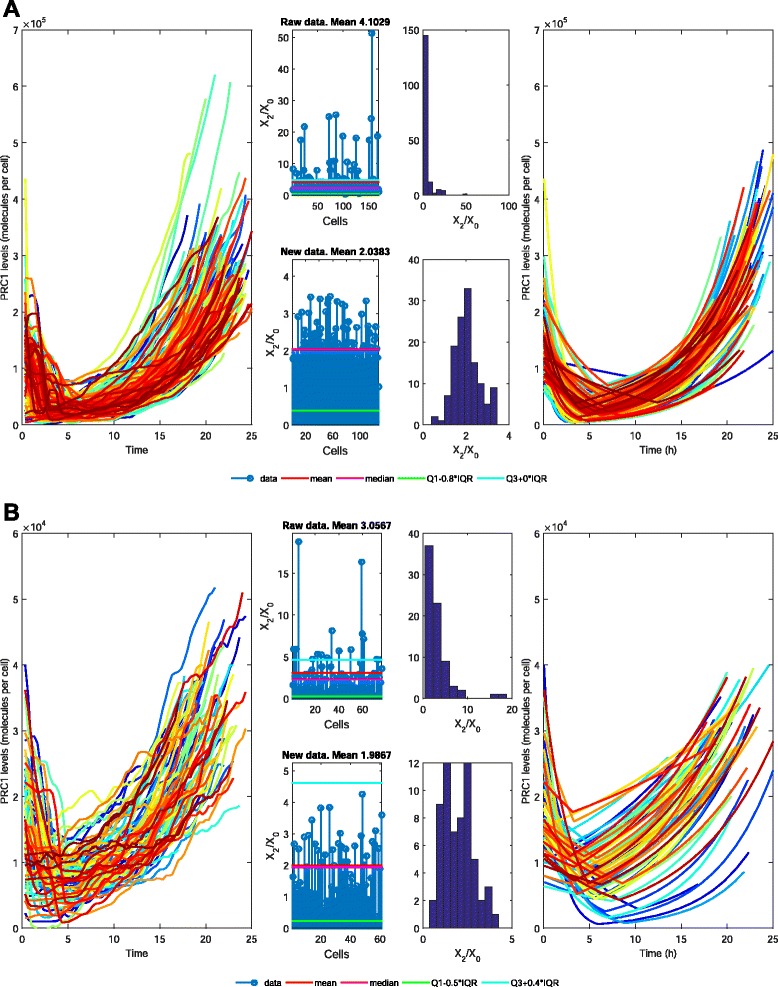
Empirical and simulated trajectories of the PRC1 protein content for the 4 data sets from reference [9]. **a** *Data set 1.* Published in the main body of [9] The composite picture includes illustration of outlier removal, empirical trajectories excluding outliers, and simulated trajectories. **b** *Data set 2.* We use it for comparison with Data set 1. (**c** and **d**) *Data sets 3 and 4*

We have at our disposal the four PRC1 data sets available from the Supplemental Data to reference [9]. Each of the data sets consists of individual-cell measurements of PRC1 contents collected at constant time intervals. The trajectories are depicted in Fig. 2, the legend of which contains the relevant details. Briefly, after division, the level of PRC1 decreases and then increases, to reach a maximum value immediately before division.

#### Detailed model description and estimation

Based on data analysis, the model entails the following detailed principles:

1. ln(*a*) depends linearly on *X*_0_ with additive Gaussian noise,
2. ln(*T*_1_) (*T*_1_ in case of Data set 2) depends linearly on ln(*a*) with additive Gaussian noise,
3. $$ {X}_1={X}_0 \exp \left(-a{T}_1\right), $$
4. ln(*b*) depends linearly on ln(*a*) and *X*_1_ with additive Gaussian noise,
5. *T*_2_ depends linearly on *T*_1_ with additive Gaussian noise,
6. $$ {X}_2={X}_1 \exp \left(b{T}_2\right), $$
7. *X*_0_ ' = *UX*_2_, *X*_0_ ' ' = (1 − *U*)*X*_2_, i.e., in multi-generation simulations, the next-generation starting protein contents are modeled using Eq. 2, where the distribution of the random variable *U* is assumed to belong to the Beta-family.

Table 1 depicts the correlations computed from the data. They indicate a strong positive correlation between ln(*a*) and *X*_0_, which supports item 1 above. Similarly, there exists a strong negative correlation between ln(*T*_1_) and ln(*a*), which supports item 2. For Data set 2, the correlation is slightly better for *T*_1_ and ln(*a*). Then, *X*_1_ can be computed from the exponential decay expression as in item 3. Further, there exists a strong positive correlation between ln(*b*) and ln(*a*), and a strong negative correlation between ln(*b*) and *X*_1_, which supports item 4. Finally, there exists a strong negative correlation between *T*_1_ and *T*_2_, which supports item 5. Then, *X*_2_ can be computed from the exponential growth expression as in item 6.

**Table 1:** Data-derived correlations between pairs of variables characterizing the PRC1 trajectories in Data sets 1–4

Data set 1									
	X_0_	X_1_	X_2_	T_1_	T_2_	T_1_ + T_2_	a	ln(a)	b
X_1_	−0.02								
X_2_	0.30	0.11							
T_1_	−0.08	0.25	−0.05						
T_2_	0.05	−0.25	0.15	−0.62					
T_1_ + T_2_	−0.03	−0.02	0.13	0.39	0.48				
a	0.24	−0.58	0.06	−0.75	0.57	−0.17			
ln(a)	0.31	−0.71	0.05	−0.76	0.55	−0.21	0.93		
b	0.05	−0.71	0.18	0.11	−0.29	−0.22	0.29	0.38	
ln(b)	0.05	−0.75	0.19	0.10	−0.27	−0.21	0.30	0.41	0.98
Data set 2									
	X_0_	X_1_	X_2_	T_1_	T_2_	T_1_ + T_2_	a	ln(a)	b
X_1_	0.06								
X_2_	0.35	0.19							
T_1_	0.22	0.13	0.00						
T_2_	0.07	−0.22	0.32	−0.42					
T_1_ + T_2_	0.26	−0.11	0.31	0.45	0.62				
a	−0.03	−0.63	−0.04	−0.59	0.24	−0.27			
ln(a)	0.10	−0.77	−0.01	−0.66	0.43	−0.12	0.87		
b	−0.02	−0.78	0.11	−0.04	−0.12	−0.14	0.63	0.60	
ln(b)	−0.01	−0.81	0.12	0.02	−0.12	−0.10	0.60	0.59	0.99
Data set 3									
	X_0_	X_1_	X_2_	T_1_	T_2_	T_1_ + T_2_	a	ln(a)	b
X_1_	0.06								
X_2_	0.26	0.29							
T_1_	0.23	0.12	0.02						
T_2_	0.09	−0.19	0.33	−0.34					
T_1_ + T_2_	0.27	−0.08	0.32	0.47	0.67				
a	0.03	−0.63	−0.10	−0.55	0.22	−0.23			
ln(a)	0.17	−0.76	−0.11	−0.61	0.38	−0.10	0.88		
b	−0.04	−0.77	0.04	−0.06	−0.15	−0.19	0.62	0.59	
ln(b)	−0.03	−0.79	0.06	0.00	−0.15	−0.14	0.58	0.57	0.99
Data set 4									
	X_0_	X_1_	X_2_	T_1_	T_2_	T_1_ + T_2_	a	ln(a)	b
X_1_	0.21								
X_2_	0.31	0.18							
T_1_	0.31	0.19	0.06						
T_2_	0.26	−0.39	0.50	−0.11					
T_1_ + T_2_	0.41	−0.25	0.48	0.44	0.84				
a	0.17	−0.58	0.03	−0.40	0.41	0.16			
ln(a)	0.23	−0.66	0.01	−0.36	0.48	0.27	0.84		
b	−0.22	−0.73	−0.07	−0.03	−0.04	−0.06	0.49	0.46	
ln(b)	−0.25	−0.79	−0.06	−0.06	0.00	−0.03	0.48	0.46	0.97

#### Detection and removal of outliers in the data

The first step was finding and removing the outlier trajectories of PRC1 protein. In all 4 data sets the individual measurements differed with respect to the dynamic of this protein. We decided that the most important is how much the amount of PRC1 increases during cell cycle. When the *X*_2_/*X*_0_, ratio was calculated, in raw data in some cells the number of protein molecules at the end of the cell cycle was up to fifty times higher than at the beginning, which is biologically unlikely. We use a modification of Tukey test to eliminate the outliers [19]. Figure 2 shows the *X*_2_/*X*_0_ ratio and distributions before and after removal of outliers.

We believe that outliers result from measurement errors. The beginning or the end of the cell cycle might have been identified incorrectly.

#### Estimation of model parameters

Following determination of the structure of the model, estimation of the coefficients of the linear relationships and the variances of noise has been accomplished using standard regression techniques [19]. Final relationships for Data set 1 are presented in Table 2. Table 3 presents the simulation-based counterparts of experimental correlations from Table 1 (Data set 1). Each cell has different values of all parameters.

**Table 2:** Regression-based coefficients for the 4 versions of the model based on Data sets 1–4, respectively

Data set 1						
	1	X_0_	ln(a)	T_1_	X_1_	ε
ln(a)	−1.600	3.9 × 10^−6^	-	-	-	N(0, 0.62)
ln(T_1_)	1.110	-	−0.514	-	-	N(0, 0.124)
ln(b)	−1.570	-	−0.100	-	−1.78 × 10^−5^	N(0, 0.07)
T_2_	19.970	-	-	−0.6564	-	N(0, 1.62)
Data set 2						
	1	X_0_	ln(a)	T_1_	X_1_	ε
ln(a)	−3.37	7.82 × 10^−5^	-	-	-	N(0,0.45)
T_1_	4.81		0.0465	-	-	N(0,2.00)
ln(b)	−1.7	-	−0.0157	-	−9.89 × 10^−5^	N(0,0.08)
T_2_	18.93	-	-	−0.75	-	N(0,2.54)
Data set 3						
	1	X_0_	ln(a)	T_1_	X_1_	ε
ln(a)	−1.75	5.01 × 10^−6^	-	-	-	N(0,0. 0.35)
ln(T_1_)	1.18		−0.349	-	-	N(0,0.15)
ln(b)	−1.8	-	−0.0151	-	−2.12 × 10^−5^	N(0,0.09)
T_2_	19.91	-	-	−0.40	-	N(0,2.53)
Data set 4						
	1	X_0_	ln(a)	T_1_	X_1_	ε
ln(a)	−2.10	1.39 × 10^−5^	-	-	-	N(0,0.485)
ln(T_1_)	1.41		−0.1123	-	-	N(0,0.11)
ln(b)	−1.9	-	−0.0437	-	−3.16 × 10^−5^	N(0,0.11)
T_2_	20.90	-	-	−0.37	-	N(0,2.16)

**Table 3:** Simulation-based correlations between pairs of variables characterizing the PRC1 trajectories in Data sets 1–4

Data set 1									
	X_0_	X_1_	X_2_	T_1_	T_2_	T_1_ + T_2_	a	ln(a)	b
X_1_	0.23								
X_2_	−0.04	0.08							
T_1_	−0.25	0.55	−0.06						
T_2_	0.16	−0.34	0.56	−0.62					
T_1_ + T_2_	−0.08	0.20	0.60	0.37	0.60				
a	0.34	−0.63	−0.20	−0.75	−0.20	−0.29			
ln(a)	0.30	−0.74	−0.09	−0.91	−0.09	−0.33	0.91		
b	−0.34	−0.90	0.09	−0.36	0.09	−0.13	0.46	0.54	
ln(b)	−0.36	−0.91	0.10	−0.35	0.10	−0.13	0.44	0.53	0.99
Data set 2									
	X_0_	X_1_	X_2_	T_1_	T_2_	T_1_ + T_2_	a	ln(a)	b
X_1_	−0.03								
X_2_	−0.28	0.33							
T_1_	0.04	−0.58	−0.42						
T_2_	−0.04	0.27	0.79	−0.48					
T_1_ + T_2_	−0.02	−0.12	0.56	0.21	0.56				
a	0.75	−0.45	−0.34	0.04	−0.34	−0.01			
ln(a)	0.77	−0.40	−0.29	0.06	−0.29	0.00	0.89		
b	0.12	−0.94	−0.31	0.54	−0.31	0.11	0.53	0.45	
ln(b)	0.00	−0.97	−0.25	0.56	−0.25	0.12	0.42	0.36	0.97
Data set 3									
	X_0_	X_1_	X_2_	T_1_	T_2_	T_1_ + T_2_	a	ln(a)	b
X_1_	0.57								
X_2_	0.16	0.25							
T_1_	−0.19	−0.03	−0.11						
T_2_	0.03	0.01	0.75	−0.17					
T_1_ + T_2_	−0.05	0.00	0.68	0.25	0.68				
a	0.31	−0.43	−0.09	−0.60	−0.09	−0.16			
ln(a)	0.31	−0.43	−0.07	−0.63	−0.07	−0.17	0.97		
b	−0.50	−0.86	0.01	0.04	0.01	0.01	0.38	0.37	
ln(b)	−0.51	−0.88	0.01	0.04	0.01	0.01	0.37	0.37	0.99
Data set 4									
	X_0_	X_1_	X_2_	T_1_	T_2_	T_1_ + T_2_	a	ln(a)	b
X_1_	0.33								
X_2_	0.01	0.31							
T_1_	−0.20	0.10	0.06						
T_2_	0.01	−0.01	0.57	−0.10					
T_1_ + T_2_	−0.05	0.02	0.58	0.19	0.58				
a	0.43	−0.59	−0.35	−0.44	−0.35	−0.10			
ln(a)	0.45	−0.60	−0.29	−0.48	−0.29	−0.10	0.93		
b	−0.34	−0.87	−0.03	−0.05	−0.03	−0.01	0.50	0.48	
ln(b)	−0.35	−0.90	−0.02	−0.05	−0.02	−0.01	0.48	0.48	0.98

**Fig. 3:**
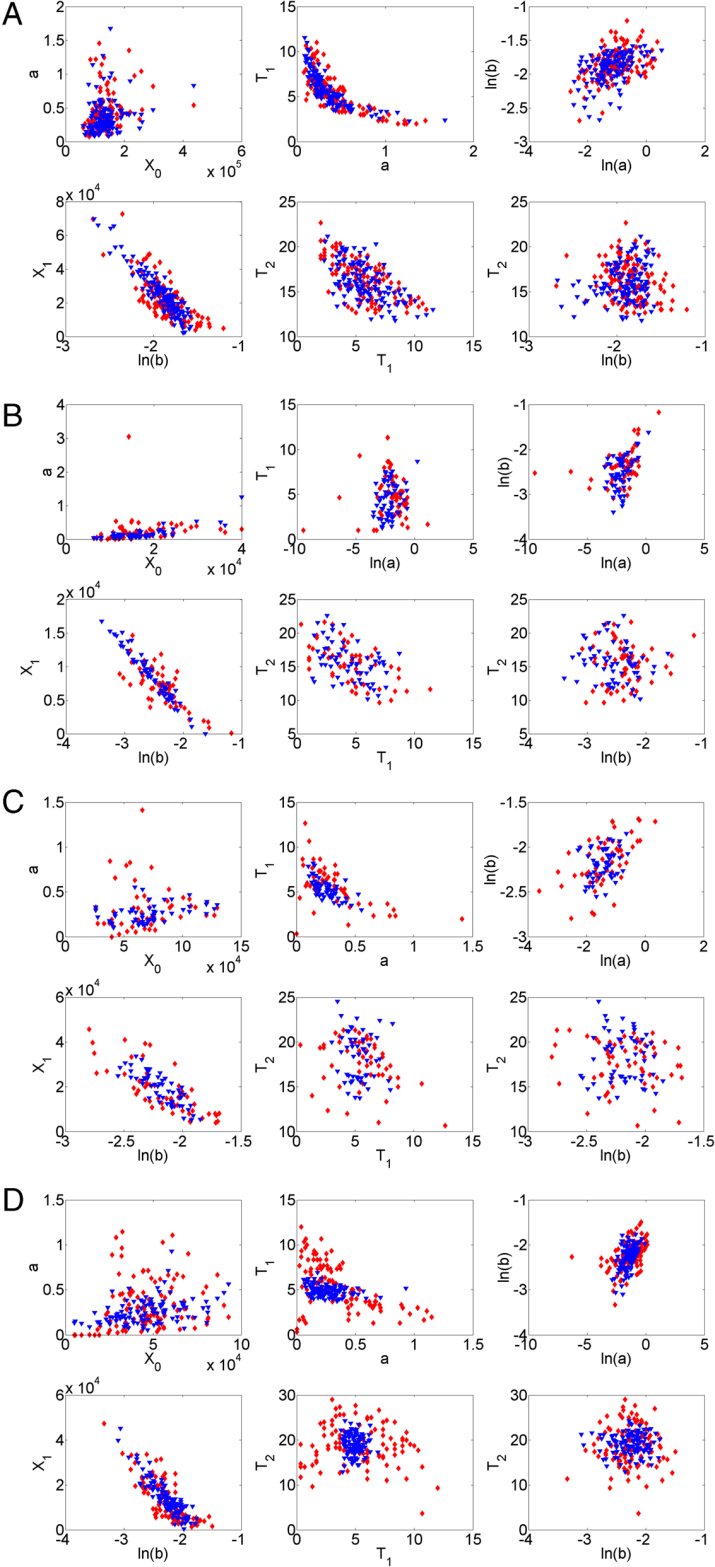
Data-based vs. simulation-based scatterplots of relationships among principal model variables for Data sets 1–4 (**a**–**d**). Symbols: data-based, red diamonds; simulation-based, blue triangles

Comparison of the scatterplots of experimental vs. model-based relationships among model variables is depicted in Fig. 3 (Data sets 1–4). Comparison demonstrates a very good agreement of experiment and model-based data, especially for Data set 1, which was used to construct the model.

We have not observed many cases where calculated parameter was negative. Nevertheless, when such a case happened we rejected the calculated parameter and draw another ε value. Effectively, this means we use Gaussian noise, conditional on nonnegativity.

As for the unequal division, the coefficient of variation of the random variable *U*, characterizing the asymmetry of division, is estimated from Eq. (4). In simulations, the amount of proteins received by daughter cells was sampled from a symmetric beta distribution, which has the variance equal to *V*(*U*) = (8*α* + 4)^− 1^. Values of parameters of beta for all data sets are depicted in Table 4.

**Table 4:** Parameters of the beta distribution of the random variable *U*, characterizing the asymmetry of division

Data set 1	
α	29.29
β	29.29
Data set 2	
α	15.79
β	15.79
Data set 3	
α	35.41
β	35.41
Data set 4	
α	25.95
β	25.95

## Results and discussion

### Simulations of population dynamics

We have run the model for a large number of cell generations, recording the PRC1 contents in all cells of the resulting pedigree, at constant time intervals. To generate the population of cells, simulation was started with a single ancestor cell. During cell division the PRC1 is unequally divided between daughter cells as described above.

**Fig. 4:**
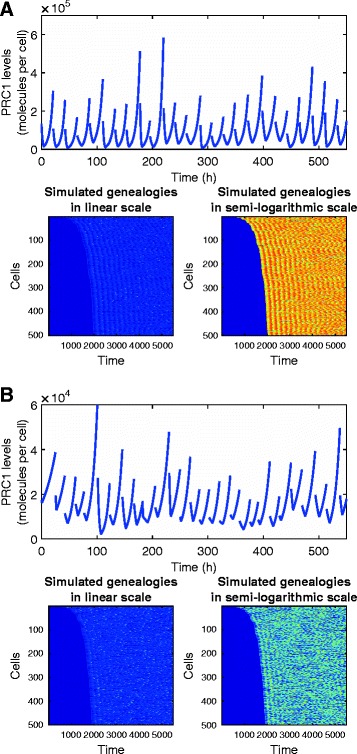
Results of long-term simulations of the model based on Data set 1 (**a**) and Data set 2 (**b**). Composite figures include: Dynamics of the time series of the PRC1 contents in a randomly chosen lineage of descendants of the ancestor cell; and color-scale depiction of the simulated genealogies in linear scale (highlighting outliers) and semi-logarithmic scale

Results of long-term simulations of the model based on Data sets 1 and 2 are presented in Fig. 4. The picture emerging from simulations of Data set 1 is that of a very well-tuned regulatory circuit that provides a stable distribution of PRC1 contents and interdivision times. Outliers, being usually particularly high values of *X*_2_ appear sporadically and are eliminated in the succeeding 1 or 2 generations. Data set 2 seems qualitatively different, with more variation in cell cycle duration.

### Simplified mathematical model explaining the cell cycle regulation

The equations of the model can be written explicitly, including the noise terms, based on the detailed model description (items 1–6 from the list earlier on), with parameter values depending on the data set, as listed in Table 2. It is instructive to consider a model stripped of noise, which very clearly shows the straightforward nature of cell cycle regulation as estimated from the data. The model will be shown to be very robust to noise introduced by asymmetric division and, at least numerically, this can be extended to any source of noise. The equations of the model without noise are as follows:

5 $$ \begin{array}{l} \ln (a)={b}_1+{b}_{10}{X}_0\\ {} \ln \left({T}_1\right)={b}_2+{b}_{21} \ln (a)\kern0.5em \left( Data\kern0.5em  set s\kern0.5em 1,\kern0.5em 3,\kern0.5em 4\right)\\ {}{T}_1={b}_2+{b}_{21} \ln (a)\kern0.5em \left( Data\kern0.5em  set\kern0.5em 2\right)\\ {} \ln \left({X}_1\right)= \ln \left({X}_0\right)-a{T}_1\\ {} \ln (b)={b}_4+{b}_{41} \ln (a)+{b}_{43}{X}_1\\ {}{T}_2={b}_5+{b}_{52}{T}_1\\ {} \ln \left({X}_2\right)= \ln \left({X}_1\right)+b{T}_2\end{array} $$

**Fig. 5:**
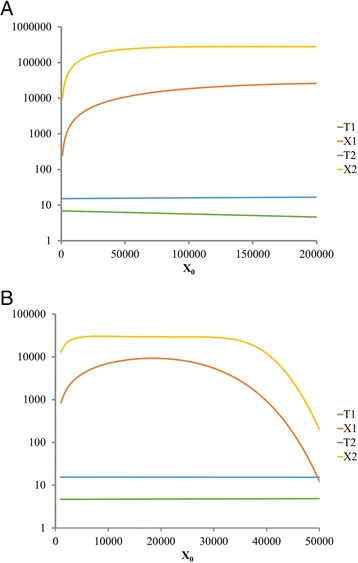
Relationships derived from the deterministic version of the model based on Data sets 1 (**a**) and 2 (**b**). Horizontal axis, *X*_0_, graphs depict variables *X*_1_, *X*_2_, *T*_1_ and *T*_2_, as functions of *X*_0_

Explicit expressions for all the variables can be found, although they are cumbersome. In particular, we obtain the functions *X*_2_ = *ϕ*(*X*_0_), *T* = *ψ*(*X*_0_), with *T* = *T*_1_ + *T*_2_, which define the Kimmel et al. model [5]. Figure 5 depicts *X*_1_, *X*_2_, *T*_1_, and *T*_2_, as functions of *X*_0_, for Data sets 1 and 2. The relationships are somewhat different in both cases; Data set 1 exhibits monotonous increasing dependence of *X*_2_ on *X*_0_, while for the Data set 2, the *X*_2_ graph attains a maximum and then decays to 0. Remarkably, *T*_1_, and *T*_2_ are practically constant as functions of *X*_0_ in both cases.

**Fig. 6:**
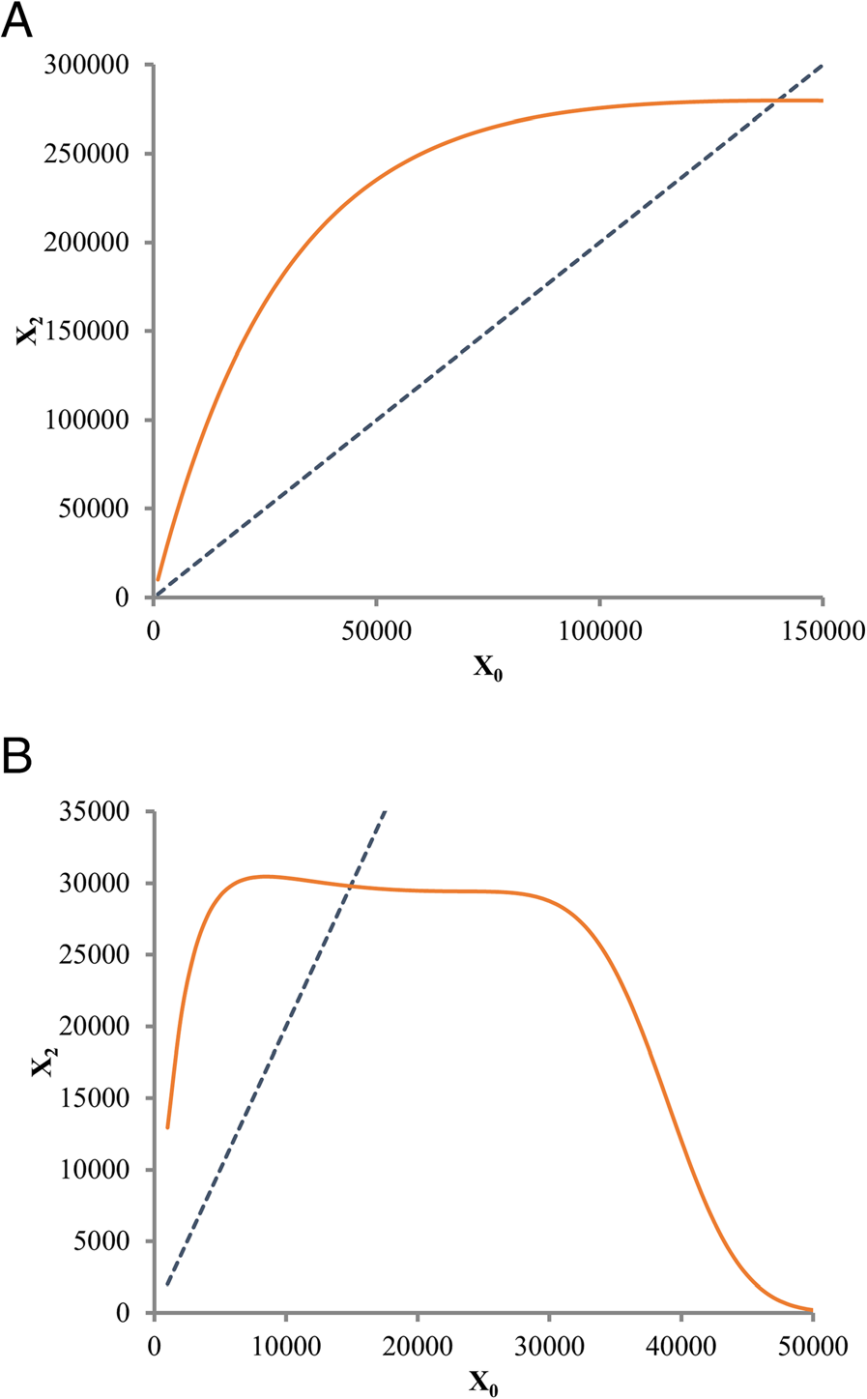
Graphical depiction of the equilibria based on the model and Data sets 1 (**a**) and 2 (**b**). Horizontal axis, *X*_0_, graph of *X*_2_ as a function of *X*_0_ (continuous line), intersected with the graph of 2*X*_0_

**Fig. 7:**
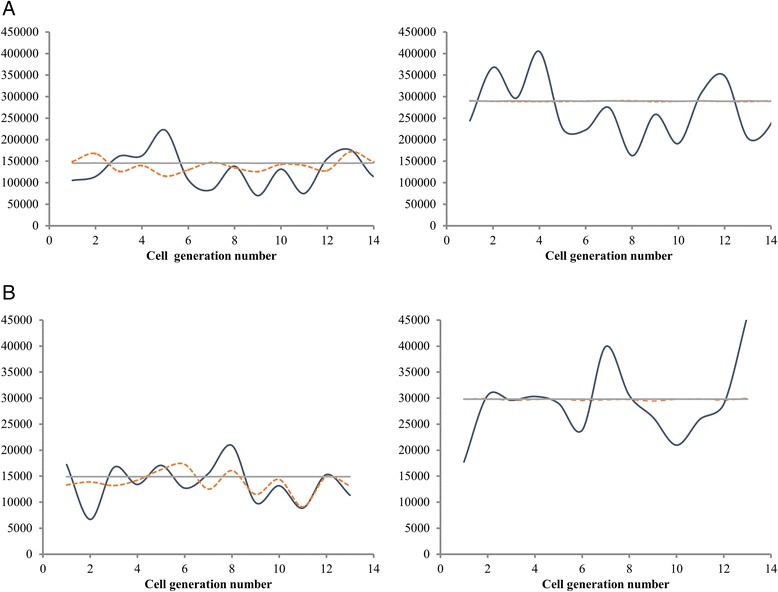
Oscillations of the PRC1 levels before and after division, generated by models based on Data sets 1 (**a**) and 2 (**b**). For several generations of a randomly chosen lineage started by an ancestral cell, series of values of *X*_0_ and *X*_2_ (interpolated by smooth lines for optical convenience) are depicted. Continuous lines, *X*_0_; dashed lines *X*_2_. For the case of a deterministic model with asymmetry of divisions being the only source of randomness, the continuous line is superimposed on the reference deterministic equilibrium

In the case of the deterministic model (5), if in addition the divisions are symmetric, the equilibrium value of *X*_0_ satisfies the equation

6 $$ {X}_0=\phi \left({X}_0\right)/2 $$

as shown in Fig. 6. However, with asymmetric division, the equilibrium is disrupted since

7 $$ {X}_0\hbox{'}=U\phi \left({X}_0\right) $$

where *X*_0_ ' is the initial PRC1 contents in the randomly chosen progeny. This results in the values of *X*_0_ oscillating from one generation to another, while the values of *X*_2_ = *ϕ*(*X*_0_) are much less affected (Fig. 7), which illustrates the efficiency of the regulatory mechanism. This is exactly the case considered in [5] and in [20] and the equilibrium distribution can be computed using methods of these papers. Finally, if the full stochastic model is used, then both *X*_0_ and *X*_2_ oscillate, since the uncertainty embedded in the model counteracts the regulatory feedback. This latter case has not been studied analytically.

### Parent-progeny and sib-sib correlations

**Fig. 8:**
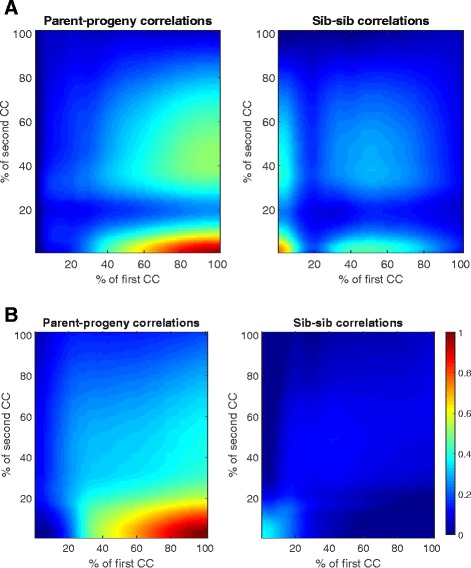
Simulation-based correlations of PRC1 protein for each time point of the cell cycle (percentage) on (**a**) Data set 1, and (**b**) Data set 2

These correlations were computed in [9] for Data set 1. We present model-based correlations in Fig. 8. They are somewhat different from those in the original paper, which however may be the question of scaling and color-coding.

## Conclusions

It is interesting and important to understand the mechanisms of epigenetic regulation in proliferating eukaryotic cells. There exist a number of models, with very strong experimental background, which explain the interplay of signaling pathways underlying the timing of cell division including stochastic effects [21]. In addition to this, there exists a very large body of literature addressing experimental relationships among cell size at birth, duration of the cell cycle and asymmetry of division. Idiosyncratically, we may mention models of Kimmel et al. [5], Dyson et al. [3], and Di Talia et al. [22]. One of these models [23] based on observations on embryonic cells led to a bimodal distribution of cell sizes in the population.

Asymmetry of division has been deemed to play a major role in generation of variability in cell populations. Various molecular mechanisms may underlie asymmetric cell division. For example, in our previous works, we use a stochastic model based on branching processes, which qualitatively describe new wave of single cell-based observation. The model, originally devised in [24] to model evolution of unstable gene amplification and then analyzed mathematically by other, is presented in Fig. 1b. We consider a set of large particles (biological cells), following a binary fission process. Each of the large particles is born containing a number of small particles (genes, proteins, viruses, organelles), which multiply or decay during the large particle’s lifetime. The arising population of small particles is then split between the two progeny of the large particle and the process continues in each of the progeny. This “division-within-division” or “branching-within-branching” occurs in various settings in cell and molecular biology. Examples include tightly regulated phenomena such as replication of chromosomal DNA, but also processes in which the number of objects produced in each biological cell is a random variable.

Recent progress in single-cell measurement techniques enabled a much more precise look at cell cycle kinetic in individual cells. We based our modeling up-to-date on the publicly available data from Alon’s laboratory. They tracked levels of a number of cell-cycle related and other proteins, some of them over a number of cell cycles and presented synthetic statistics for some of them [9].

Methodologically, we developed a relatively simple model allowing peeling off layers of stochasticity, related to intermediate stages in PRC1 regulation. The initial variability of PRC1 is party cancelled by resetting it to a low level and then increasing its contents until division. We do not know how the timing of the minimum of PRC1 is related to the cell-cycle phases; this is an interesting question in itself. The model, when stripped of stochasticity except for asymmetry of division, reduces to the old model of Kimmel et al. [5], which has been completely characterized mathematically [20] using tools of the operator semigroup theory.

It may be mentioned that in another paper, Arino and Kimmel [25] analyzed a model, which included more stochastic elements than the original model in ref. [5]. In that model, in addition to the asymmetric division, the time the progeny cell spends in the cycle is a random variable with conditional distribution density given its birth size. It has been demonstrated that this approach, originating in the theory of branching processes, is essentially equivalent to the more typical (at the time) formulation in the form of a transport partial differential equation with a nonlocal feedback through boundary condition. For further discussion, see ref. [25].

In the current paper, we used a mathematical model to reproduce experimental trajectories of the PRC1 protein published in [9] and extend the results to model long-range dynamics of the cell population. The main question we address is whether the regulatory feedbacks deduced from single cell cycle data provide epigenetic regulation of cell characteristics in long run. PRC1 protein is regulated by the cell cycle. This protein is absolutely required in cytokinesis, without it cell cannot divide to form two daughter cells [26]. PRC1 is a good candidate because of its role in setting timing of division. Findings of the current paper include tight regulation of the cell cycle (particularly the timing of the cell cycle) even that PRC1 is only one of the players in cell dynamics. Understanding that association, even close, does not necessarily imply causation, we consider this an interesting and important result.

In recent publications authors analyzed single-cell data. Authors of the first paper [27], used the Fucci system (the first marker indicates G0/G1 phases, and the second one the S/G2/M phases) to calculate the length of the cell cycle and the cell cycle phases. They calculated correlations between parent-progeny (no correlation), siblings and cousins (high correlations) cell cycle lengths. Obtained results can be explained by circadian clock control over the mammalian cell cycle in cell. Dynamic of the two Fucci makers was not analyzed, so it is difficult to compare ref. [28] with our work, which is mainly focused on protein dynamics.

In another paper [28] authors analyzed what impact on cell signal response intrinsic and extrinsic noise has and how cells can eliminate variability causes by extrinsic noise. They performed single-cell measurements of three key signal pathways: extracellular signal-regulated kinase, calcium and nuclear factor kappa-B. Again, this paper has a different focus.

Also recently, single-cell expression of cell cycle regulators was analyzed in ref. [29], but the authors explained variability in cell cycle length in the terms of a mammalian clock control. To confirm their theory they proposed a simple linear mathematical model. We used a more parsimonious paradigm of correlation and regression methods to predict what directed influences on a cell cycle are caused by number of protein. The novelty of the present study is the combination of single cell experimental data, correlation analysis and mathematical modeling of individual cell dynamics.

## Reviewers’ Comments

First of all we would like to thank the referees for their comments and suggestions that were addressed as follows:

### Reviewer’s report 1: Prof. Ollivier Hyrien, University of Rochester

This manuscript deals with studying the contribution of PRC1 to the regulation of the cell cycle. A mathematical framework is proposed that describes (1) the dynamics of PRC1 during the cell cycle, (2) the random allocation of the protein at division, and (3) cell kinetics. The model is interesting and developed based on an earlier stochastic model proposed by Kimmel and colleagues (1984). An application of this model is presented in which the authors analyze data on the protein dynamics in H1299 non-small cell lung cancer cell lines published by Cohen et al. (2009). Simulations indicate that the model achieves a good description of experimental data.

Authors’ response: *Thank you for a positive overview.*

Page 5, Eq. 3. Are the parameters a and b constrained to be positive? Are they random (i.e., cell-specific) or are they identical across cells?

Authors’ response: *Parameters a and b are always positive. Every cell has different values of these parameters. Based on initial number of molecules (X*_0_*) we use linear regression to calculate* log*(a)*

$$ log(a) = {b}_1 + {b}_{10}{X}_0 + \varepsilon $$

*b*_1_*, b*_10_*and* ε *are as described in* Table 2*(similar principles are used to estimate* log(*b*)*). They differ among data sets.*

*As you can see, we do not have to constrain a and b to be positive.*

*We add additional information on page 5.*

Page 6: “T_2_ depends linearly on T_1_ with additive Gaussian noise”. Since T_2_ is a duration, perhaps what is meant here is simply that T_2_ is linearly associated with T_1_, without making any distributional assumption about the noise. This would preserve the positivity of T_2_.

Authors’ response: *The noise term is needed to obtain agreement with the data. We have not observed many cases where calculated T*_2_*was a negative value. Nevertheless, when such case happened we rejected calculated T*_2_*and drew another* ε *value. Effectively, this means we use Gaussian noise, conditional on nonnegative T*_2_*.*

Page 7: “… at the end of the cell cycle was up to 50 times higher than at the beginning, which is biologically unlikely”. Is it the beginning of the cell cycle of the beginning of the accumulation phase. Also, could the authors comment briefly on possible explanations for why the ratio *X*_2_/X_1_ was so high in some cells? For example, could this be due to the nonlinearity of the relationship between fluorescence intensity and number of molecules, or to measurement errors?

Authors’ response: *Apologies for misprint. We calculate the X*_2_*/X*_0_*ratio, so the ratio between number of molecules at the beginning and at the end of the cell cycle.*

*We believe that outliers result from measurement errors. The beginning or the end of the cell cycle might have been identified wrongly. The measurements were performed before 2009, when cell tracking was less well developed.*

Page 7: Was parameter estimation performed on the trajectory of protein concentration for each cell individually? Pages 8–9: In running model simulations, were the random times T1 and T2 assumed to follow specific distributions?

Authors’ response: *Parameters in equations (5) were estimated using data from all cells. In this paper, we have not made any distributional assumptions, except for Gaussian distributions of noise terms.*

Page 9: The dependence structure induced by the assumed mechanism of protein dynamics is an interesting feature of the model. Could the authors elaborate on the comparison between model-based correlations and those obtained from experimental data?

Authors’ response: *Model-based (*Table 3*) and data-based correlations (*Table 1*) are in good agreement, particularly when their absolute values are high. The agreement is best for Data set 1, which was used as a reference to create the model.*

### Reviewer’s report 2: Prof. Anna Marciniak-Czochra, University of Heidelberg 

Authors consider a mathematical model of epigenetic control of the cell cycle, taking into account stochastic effects and resulting heterogeneity of cell population. Such models have been conceived in the past (see Kimmel’s own model published in 1984), but have been largely abandoned for the lack of precise measurements of biomolecules at a single-cell scale. The topic has become important in part because of progress in quantification and in part because of recent emphasis on epigenetic controls of the cell cycle.

The model employs publicly available data from Alon’s laboratory, in particular, single-cell trajectories of the PRC1 protein involved in cell cycle controls. Using a multistep estimation procedure, authors successfully build a model that reconstructs the stochastic dynamics at the single-cell level and marginal and joint distributions of most of the meaningful parameters. Authors also demonstrate that if stripped of various layers of dynamics, the model can be reduced to Kimmel et al. 1984 model of cell cycle regulation. Also, it leads to cell population homeostasis when run for extended times.

There are some interesting points that the authors should address before the paper becomes suitable for Biology Direct:

The model in its mathematical framework considers a factor (protein) that may be an active regulator of the cell cycle. It might be worthwhile to discuss if PRC1 qualifies as such factor.

Authors’ response: *PRC1 protein is not an active regulator of the cell cycle per se, but it is regulated by the cell cycle. This protein is absolutely required in cytokinesis, without it cell cannot divide to form two daughter cells. More precisely, the central spindle bundle is not formed and this prevents the final abscission event* [26]*. We think that because of a strong correlation to cell-division events, PRC1 protein qualifies to be used in our model.*

Authors provide simulations of the long-term dynamics of the model under variable levels of stochasticity. What is missing is a discussion of mathematical results that might be relevant for establishing long-term homeostasis of the model.

Authors’ response: *Recently, single-cell expression of cell cycle regulators was analyzed in ref.* [29]*, but the authors explained variability in cell cycle length in the terms of a mammalian clock control. To confirm their theory they proposed a simple linear mathematical model.*

*We used a more parsimonious paradigm of correlation and regression methods to predict what directed influences on a cell cycle are caused by number of protein. The novelty of the present study is the combination of single cell experimental data, correlation analysis and mathematical modeling of individual cell dynamics.*

Finally, recently, there have been a number of new papers published, which either involve similar models, or show new techniques for obtaining data at single-cell level (see Nature 2015, 519, 468–471, or Science 2014, 346, 1370–1373). Enhanced discussion of these models, compared to the model in the present manuscript, is desirable.

Authors’ response: *In these two publications authors analyzed single-cell data. Authors of the first paper* [27]*, used the Fucci system (the first marker indicates G0/G1 phases, and the second one the S/G2/M phases) to calculate length of cell cycle and cell cycle phases. They calculated correlations between parent-progeny (no correlation), siblings and cousins (high correlations) cell cycle lengths. Obtained results can be explained by circadian clock control over the mammalian cell cycle in cell. Proteins dynamic of two makers was not analyzed, so it is hard to compare that with our work, which is mainly focused on protein dynamics.*

*In the second paper* [28] *authors analyses what impact on cell signal response have intrinsic and extrinsic noise and how cell can eliminate variability causes by extrinsic noise. They performed single-cell measurements of three key signal pathways: extracellular signal-regulated kinase, calcium and nuclear factor kappa-B.*

### Reviewer’s report 3: Prof. Alberto d’Onofrio, International Prevention Research Institute

In this computational epigenetics works the authors investigate how the regulation of the PRC1 protein content influence the long term behaviour of a cellular population.

The general topic of how epigenetic changes impact on a population is one of the most important of molecular biology, and it is at the interface between systems biology and population dynamics. I think that the idea of the manuscript is very good, the work is well written (apart an important minor detail) and the results are of interest.

Authors’ response: *Thank you for a very positive comment.*

I recommend its acceptance upon a minor but important change is implemented. The changes concerns that fact the in this work the authors do not provide enough mathematical details of the original model of unequal divisions by Kimmel et al. (1984), which makes the paper more difficult to be read for those who, differently form myself, did not read it.

Authors’ response: *We included additional information about the model of Kimmel* et al. *1984.*

I suggest of inserting in the full text the supplemental figure S1.

Authors’ response: *We included Figure S1 in the main body of the paper (currently* Fig. 3*)*

There is some typos. For example in the abstract “we follow the philosophy of a 1984 model by Kimmel model”

Authors’ response: *Apologies for the typos. We corrected all of them.*

## Abbreviations

PRC1: Protein Regulator of cytokinesis 1
CDKs: Cyclin-dependent kinases
H1299: Human non-small cell lung carcinoma cell line
